# Acute Microvascular Changes after Subarachnoid Hemorrhage and Transient Global Cerebral Ischemia

**DOI:** 10.1155/2013/425281

**Published:** 2013-03-25

**Authors:** Michael K. Tso, R. Loch Macdonald

**Affiliations:** Division of Neurosurgery, St. Michael's Hospital, Labatt Family Centre of Excellence in Brain Injury and Trauma Research, Keenan Research Centre of the Li Ka Shing Knowledge Institute of St. Michael's Hospital, Department of Surgery, University of Toronto, Toronto, ON, Canada M5B 1W8

## Abstract

Subarachnoid hemorrhage and transient global cerebral ischemia result in similar pathophysiological changes in the cerebral microcirculation. These changes include microvascular constriction, increased leukocyte-endothelial interactions, blood brain barrier disruption, and microthrombus formation. This paper will look at various animal and preclinical studies that investigate these various microvascular changes, perhaps providing insight in how these microvessels can be a therapeutic target in both subarachnoid hemorrhage and transient global cerebral ischemia.

## 1. Introduction

Subarachnoid hemorrhage (SAH) is a type of hemorrhagic stroke, most commonly caused by a ruptured intracranial aneurysm. At the time of aneurysm rupture, blood pours into the subarachnoid space, and the intracranial pressure (ICP) inside the rigid calvarium increases sharply, causing a corresponding decrease in cerebral blood flow (CBF). The patient's clinical presentation on arrival to the hospital can depend on the degree and duration of this initial global cerebral ischemia. 

Patients with aneurysmal SAH may develop angiographic vasospasm and delayed cerebral ischemia (DCI) with onset 3–12 days after the initial rupture [1]. DCI may or may not be accompanied by large artery vasospasm as seen with vascular imaging [2]. A multicenter randomized clinical trial has not shown improvement in neurologic outcome despite ameliorating the delayed large artery vasospasm [3]. Whether this is due to efficacy of rescue therapy in the placebo groups or drug toxicity abrogating beneficial effects in the clazosentan groups has not been resolved. Nevertheless, as a result of these results, research in SAH has also investigated early brain injury and acute microvascular changes [4]. Nimodipine, an L-type calcium channel antagonist, is the only pharmacologic agent that has been shown to consistently improve neurologic outcomes in clinical trials of patients with SAH [5]. 

Similarly, cardiac arrest (CA) results in global cerebral ischemia that is transient in clinically relevant cases, since if cardiac function is not restored, the situation is of pathological interest only. Other causes of transient global cerebral ischemia (tGCI) include asphyxia, shock, and complex cardiac surgery [6]. The clinical presentation depends on the duration of cardiac arrest and time to initiating cardiopulmonary resuscitation. After global cerebral ischemia from SAH or tGCI, a cascade of molecular events occurs, resulting in variable degrees of brain injury and cerebrovascular changes.

Global cerebral ischemia in postcardiac arrest has also been studied extensively for many decades in various animal models. Other than early induced mild hypothermia [7, 8], clinical translation of neuroprotective strategies and therapeutics has largely been unsuccessful.

The study of the microcirculation after tGCI and SAH remains a difficult undertaking, but this strategy of study may reveal potential therapeutic targets and new insights into disease pathophysiology. The purpose of this paper is to look at relevant animal and preclinical studies investigating acute microvascular changes (within the first 48 hours) occurring after either SAH or tGCI. Cerebral microvessels may be defined as vessels less than or equal to 100 micrometers in diameter [9]. Animal studies of focal ischemia or studies focused on the large cerebral vessels (i.e., circle of Willis arteries, basilar artery, etc.) are not included in this paper. While we acknowledge that tGCI may occur in a large heterogeneous group of disorders (i.e., traumatic brain injury, intracerebral hemorrhage, etc.), we have chosen to focus solely on tGCI secondary to cardiac arrest or mechanisms mimicking cardiac arrest, such as extracranial arterial occlusion. After providing an overview of various animal models and general trends in cerebral hemodynamics after SAH and tGCI, we provide an in-depth review of studies investigating specific microvascular changes that occur in these two conditions: (1) microvascular constriction; (2) increased leukocyte-endothelial cell interactions; (3) blood brain barrier (BBB) breakdown; and (4) platelet aggregation and microthrombosis.

## 2. Animal Models

There are numerous animal models that attempt to mimic the clinical conditions of SAH or tGCI. Large (nonhuman primates, cats, dogs, and pigs) and small animals (mice, rats, gerbils, and rabbits) may be used. It is important to take into consideration that experimental results may vary depending on the animal model used. 

Techniques used to produce SAH include endovascular perforation, blood injection, artery avulsion or puncture, and clot placement. For example, the endovascular perforation model of SAH in the mouse may have more physiologic resemblance to the actual clinical scenario of a ruptured intracranial aneurysm, but the amount of blood in the subarachnoid space is quite unpredictable from animal to animal leading to increased variability in the results. The injection model of SAH (cisterna magna or prechiasmatic cistern) in the mouse provides the ability to control the amount of blood introduced into the subarachnoid space, but may not produce as dramatic rise in ICP compared to the endovascular perforation model, depending on the amount injected. As a result, the degree of global cerebral ischemia seen after SAH may not be as severe in the blood injection model as reflected by the overall lower mortality rate compared with the endovascular perforation model [10, 11]. A detailed review of various animal models of SAH has been published previously [12]. The type of SAH model utilized must be taken into account when interpreting experimental results.

Similarly, there are a large variety of animal models and techniques used to study tGCI. These techniques include cardiac arrest/asphyxia, thoracotomy with clamping of the aorta and great vessels, bilateral common carotid artery and vertebral artery (4 vessel) occlusion, and isolated bilateral common carotid artery occlusion. The severity of the ischemia depends on the technique used to produce ischemia, the type of animal, and even the strain of an animal species. For example, most gerbils are known to lack posterior communicating arteries that connect the forebrain and hindbrain circulations. Thus, bilateral common carotid artery occlusion produces very severe forebrain ischemia in gerbils [13]. However, in mice, the presence or absence of posterior communicating arteries varies depending on the strain used. BALB/C mice had larger infarct sizes and were more likely not to have posterior communicating arteries compared with BDF and CFW mice after concomitant ipsilateral common carotid artery and middle cerebral artery occlusions [14]. Also, the duration of ischemia and reperfusion can vary significantly between studies. A comprehensive review of available animal models of tGCI has been published [15]. Again, interpretation of study results must take into account the specific model of tGCI utilized.

## 3. Cerebral Hemodynamic Changes

After SAH, the ICP increases as a result of new subarachnoid blood occupying volume in the fixed intracranial space, with a corresponding decrease in cerebral perfusion pressure (CPP). There are no data on ICP during de novo aneurysm rupture in humans; but during rebleeding, the ICP frequently rises substantially [16]. The ICP may rise as high as the diastolic blood pressure and last for several minutes. Since not all patients go unconscious at the time of SAH, this only occurs in a subset of clinical cases. During this period, there may be a transient absence of forward CBF [17]. The mean arterial pressure (MAP) typically increases to partially compensate, but this change does not adequately restore CPP. The ICP then returns to normal or slightly supranormal levels over the course of less than an hour [17]. In a rat endovascular perforation model, CBF, which initially drops sharply to 20% of baseline flow, begins to slowly rise and then stabilizes at a level below the baseline [18]. The magnitudes of the initial drop in CBF and increase in ICP are related to the amount of subarachnoid blood [19]. If the ICP remains persistently high after SAH, then CBF does not recover and the animal dies [17].

In tGCI induced by either temporary cardiac arrest or four-vessel occlusion, there is negligible forward blood flow in the cerebral circulation. With temporary bilateral common carotid artery occlusion causing severe forebrain ischemia, the reduction in CBF is more variable depending on the intracranial collateral circulation, specifically the presence and patency of the posterior communicating arteries. Unlike in SAH, experimental models of tGCI do not produce a dramatic increase in ICP [20]. Upon reperfusion, there are two cerebrovascular response patterns seen. The first pattern is the “no-reflow phenomenon,” which is characterized by decreased tissue perfusion upon subsequent intra-arterial injection of contrast or dye after an initial period of ischemia [21]. Although the no-reflow phenomenon is more commonly discussed in the context of coronary artery occlusion [22], the term was probably first used by Ames et al., in experiments involving the cerebral circulation in rabbits undergoing tGCI [23]. This phenomenon has been confirmed in other studies [24, 25]. The second pattern is postischemic reactive hyperemia followed by delayed hypoperfusion [21]. 

Experimental SAH and tGCI both result in impaired global CBF. However, in SAH, acute cerebral ischemia is secondary in part to high ICP, which is not present in tGCI, although other mechanisms may reduce CBF after ICP declines in SAH. Also, in tGCI, reperfusion involves restoring blood flow much like an “on” switch, whereas in SAH models, reperfusion is a much more gradual process as the ICP normalizes.

## 4. Microvascular Changes in Subarachnoid Hemorrhage

### 4.1. Microvascular Constriction

Although, earlier research focused more on delayed large vessel vasospasm in SAH, it is also known that acute microvessel constriction occurs. Topical application of blood onto the cortical surface of anesthetized guinea pigs revealed vasoconstriction of pial vessels [26]. Such constriction was reversed acutely by topical application of the alpha adrenergic blocker, phenoxybenzamine, and prevented by the beta-adrenergic blocker, propranolol [26]. It appears that acute vasoconstriction occurs predominantly in the arterioles and not the venules. In an endovascular perforation model of SAH in mice, pial surface microvessels observed with *in vivo* fluorescence microscopy demonstrated unchanged venular diameter but approximately 70% of arterioles constricted acutely (3–6 hours) and persisted even at 72 hours after SAH [27]. Smaller arterioles had more vasoconstriction than larger arterioles. Pial vessels constricted as early as 5 minutes after injection of hemolyzed erythrocytes into the cisterna magna of rats, and this persisted for at least 2 hours [28]. *In vivo* monitoring also revealed decreased blood flow in the arterioles as well as the venules. Erythrocytes take time to lyse after SAH, so the time course after injection of hemolyzed blood may not be the same as after actual SAH. Using a prechiasmatic SAH model in mice, Sabri et al. found an increased degree of vasoconstriction in the microvessels (10–20 micrometers in diameter) as well as increased overall wall thickness at 48 hours after SAH, as determined by electron microscopy [29]. In these experiments, the location of the microvessel constriction appeared to strongly correlate with regional distribution of brain injury and neuronal apoptosis [29]. 

In addition to constriction, arterioles also have been shown to demonstrate altered reactivity acutely after SAH and specifically to have impaired vasodilation. In an endovascular perforation model of SAH in rats, cortical surface pial arteriolar vasodilation in response to either topical adenosine or sodium nitroprusside was significantly impaired after SAH, but CO_2_ reactivity was unaffected [30]. In addition, pial arteriolar vasodilation, which is typically seen in response to sciatic nerve stimulation, was attenuated during the first 3 days after SAH but returned to control levels by 4 days [30]. Cortical arterioles also demonstrated increased constriction in response to endothelin-1 20 minutes after injection of autologous blood into the cisterna magna injection of rats [31].

Ultrastructural changes in the walls of microvessels are also observed in experimental SAH. In an endovascular perforation model of SAH in rats, electron microscopy revealed partially collapsed capillaries with swollen astrocyte foot processes and small luminal protrusions emanating from the endothelial cells [32]. These changes occurred at least 1 hour after SAH. The significance of these luminal protrusions is unclear.

### 4.2. Leukocyte-Endothelial Interactions

Leukocyte adhesion to the microvessel wall may contribute to microvascular injury. In inflammatory conditions, the cerebral microvasculature increases the expression of endothelial adhesion molecules that attract and bind leukocytes, such as intercellular adhesion molecule-1 (ICAM-1), vascular adhesion molecule-1 (VCAM-1), P-selectin, and E-selectin [33]. With leukocytes rolling and then adhering to the microvessels, they can then traverse the luminal wall and enter the brain parenchyma by the process of diapedesis [34]. Neutrophils and macrophages may then cause direct neuronal injury [6].

After SAH induced by prechiasmatic blood injection in mice, there was a significant increase in endothelial cell membrane expression of P-selectin, but no difference in cytosolic P-selectin expression [29]. Although leukocyte adhesion was not specifically addressed in this study, the increase in P-selectin expression appeared to colocalize to regions with increased microthrombi burden [29]. Neutrophils appear to contribute to early microvascular injury after SAH. In an endovascular perforation model of SAH in rats, neutrophils were found to adhere to the cerebral microvasculature as soon as 10 minutes after SAH [35]. An inhibitor of neutrophil function, pyrrolidine dithiocarbamate (PDTC), decreased neutrophil accumulation in the parenchyma despite an increase in adherent neutrophils to the cerebral vasculature, meaning that neutrophils had impaired ability to undergo diapedesis [35]. In contrast, pharmacologic reduction of neutrophils (with vinblastine or antipolymorphonuclear serum) decreased both neutrophil adherence to cerebral microvessels and penetration into the brain parenchyma but increased subsequent bleeding. The treatments in this study also decreased collagenase activity and maintained the integrity of the BBB.

Intravital microscopy showed a progressive increase in the number of rolling and adherent leukocytes to venules at 30 minutes, 2 hours, and 8 hours after SAH induced by endovascular perforation in mice [36]. This was not seen after cisternal injection of blood, demonstrating the difference in results that can occur depending on the animal model used and suggesting a role for tGCI in the findings, since tGCI is more prominent in SAH induced by endovascular perforation compared to cisternal blood injection. Some mice were treated with a monoclonal antibody against P-selectin immediately after SAH, and this decreased leukocyte rolling and adhesion [36]. It is not clear based on preclinical SAH studies whether leukocyte plugging of microvessels as a result of increased adherence to the luminal wall is significant enough to cause ischemia in itself.

### 4.3. Blood Brain Barrier Disruption

Subarachnoid hemorrhage is believed to induce inflammatory states in the brain. Inflammatory mediators (cytokines including IL-1*β*, IL-6, TNF-*α*, and oxidative damage from neutrophils and macrophages) may result in direct damage to the microvasculature, resulting in damage to the BBB [37]. The BBB maintains an exclusive intraparenchymal compartment for the brain, separate from the circulating blood. Unlike in the systemic microcirculation, the cerebral microvessels have endothelial cells with tight junctions to prevent passage of micro- and macromolecules from the blood into the brain interstitial environment [38]. There is a lack of fenestrations between cerebral endothelial cells, which means that molecules or cells that enter the parenchyma from the microvessel lumen must migrate through the polarized endothelial cell itself. There also may be reduced pinocytosis in cerebral endothelial cells. A basal lamina embedded in an extracellular matrix encircles the endothelial cells, and this is then covered by foot processes of local astrocytes. The cerebral endothelial cell, astrocyte, and neuron form the so-called neurovascular unit [39]. Damage to the integrity of the BBB can result in brain edema and brain injury [6]. 

There are several preclinical studies that suggest that there is disruption of the BBB after SAH. The time course of disruption, the magnitude, and to what molecules the BBB is disrupted to after SAH are not fully investigated. In an endovascular perforation model of SAH in rats, there was increased BBB permeability as determined by leakage of Evan's blue dye [40]. The BBB disruption was associated with an increase in brain edema, worse neurological deficit, and mortality. A pan-caspase inhibitor (z-VAD-FMK) administered 1 hour before and 6 hours after SAH prevented BBB disruption (measured by immunoglobulin extravasation) and decreased brain edema. Although SAH caused endothelial cell apoptosis in the basilar artery, endothelial cells of the microvasculature were not assessed. In a cortical SAH model in rats, significant impairment of the BBB as determined by Evan's blue dye extravasation was observed after SAH [41]. Furthermore, in spontaneously hypertensive rats with SAH, there was more BBB disruption compared with normotensive rats with SAH [42]. In a cisterna magna injection model of SAH in rats, the time course of BBB breakdown, assessed by Evan's Blue dye extravasation, was studied [43]. The BBB breakdown began 36 hours, peaked 48 hours, and resolved 3 days after SAH. In an intracisternal SAH model in cats, the authors did not observe BBB breakdown 30 minutes after SAH [44]. Cats subjected to arterial hypertension alone demonstrated regions of BBB breakdown, whereas animals subjected to arterial hypertension after SAH did not show BBB breakdown. This protective effect of hypertension conflicts with other studies [41].

Animal studies have investigated mechanisms by which SAH may compromise the BBB. Various matrix metalloproteinases (MMPs) are capable of breaking down the basal lamina and the associated extracellular matrix surrounding the endothelial layer [45]. This may lead to blood extravasation, associated edema, and brain injury. Sehba et al. studied the integrity of the microvasculature in an endovascular perforation model of SAH in rats [45]. There was decreased immunoreactivity to type IV collagen in the microvessel basal lamina with corresponding increased levels of MMP-9 expression starting at 3 hours, peaking at 6 hours, and subsequently resolving by 48 hours after SAH. These changes were not observed at 10 minutes or 1 hour after SAH.

Extracellular matrix metalloproteinase inducer (EMMPRIN, also known as collagenase stimulatory factor, basigin, CD147, or human leukocyte activation-associated M6 antigen), is a cell surface protein that can stimulate production of MMPs [46]. Inhibition of EMMPRIN with a monoclonal antibody against it decreased brain edema 24 hours after endovascular perforation SAH in rats [46]. Brain edema was maximal at 24 hours after SAH and declined thereafter in this model [46]. In another study, using the endovascular perforation model of SAH in rats, the tight-junction protein occludin in endothelial cells and collagen type IV in the basal lamina were decreased at 24 hour after SAH [47]. Electron microscopy confirmed disruption of the endothelial tight junctions and increased spaces between endothelial cells. The investigators found that p53 colocalized with the proinflammatory transcription factor nuclear factor *κ*B (NF-*κ*B) and MMP-9, which in turn could degrade occludin [47]. Because a selective p53 inhibitor decreased microvascular damage, the authors concluded that p53 is an important factor in BBB disruption.

The direct damage to the microvasculature after SAH may in part be due to reactive oxygen species produced by inflammatory cells. In a cisterna magna injection SAH model in rats, a hydroxyl free radical scavenger, when administered within 12 hours of SAH, decreased BBB permeability at 48 hours as determined by Evan's Blue dye extravasation [48].

### 4.4. Platelet Aggregation and Microthrombosis

In SAH, clot formation in the microcirculation could occur as a result of platelet aggregation and then embolization or propagation from the original bleeding site, which would be the rupture point in the intracranial aneurysm clinically. In experimental studies, this feature of active bleeding is a component of the endovascular perforation model but not the injection models. However, arterial injury and active bleeding do not seem to be the only initiator of platelet aggregation, since microthrombi are formed even in the injection animal model of SAH in which there is no vessel rupture [29]. Also, SAH predisposes to the formation of microthrombi, as rats undergoing a prechiasmatic injection model of SAH were found to be hypercoagulable [49].

Platelet aggregates are seen in the cerebral microvasculature as early as 10 minutes after SAH induced by endovascular perforation in rats [50]. The total microclot burden peaked at 24 hours, but fully resolved by 48 hours. In another study using the same model of SAH, platelet aggregates were associated with microvessels that were poorly perfused [51]. In addition, there was breakdown of the collagen IV component of the basal lamina [52]. Platelets, upon activation, can release proteases such as MMP-9 that can digest collagen IV in the basal lamina. In fact, platelets could be seen on the abluminal side of cerebral endothelial cells and in the local parenchyma by 10 minutes after SAH, with large numbers of platelets seen in the parenchyma by 24 hours [52]. The investigators suggest that platelet aggregates may initiate or cause local endothelial cell injury, damage the BBB, and allow the extravascular escape of macromolecules and cells [51]. Sabri et al. found microthrombi throughout the mouse brain at 48 hours after the prechiasmatic injection of blood in mice [29]. These findings occurred later after SAH than demonstrated by some prior studies and in a model that has less global ischemia than the endovascular perforation model. The microclots appeared in about one-third of the constricted microvessels but in none of the normal microvessels. Also, the more severely constricted the vessel, the more numerous the microthrombi. There was a strong correlation between presence of microclots and regional brain injury.

The importance of the microthrombi to brain injury and outcome in experimental SAH was suggested in an endovascular perforation model of SAH in mice [53]. The number of microthrombi decreased upon administration of a mutant thrombin-activated urokinase-type plasminogen activator, and this correlated with decreased mortality. Platelet aggregates in SAH also adhered to leukocytes that were adherent to the walls of microvessels [36]. 

## 5. Microvascular Changes in Transient Global Cerebral Ischemia

### 5.1. Microvascular Constriction

In tGCI, the microvessels undergo significant changes in diameter during the global ischemia and then also during reperfusion; these changes affect CBF. However, reviews of the studies reveal inconsistent results. In a study by Pinard et al., a 4-vessel occlusion model of tGCI in rats was used to study *in vivo* changes of the surface pial microvessels [54]. During the 15 minutes of cerebral ischemia, arteriolar diameter transiently increased and then decreased. Cerebral autoregulation may explain this transient arteriolar vasodilation. Administration of 7-nitroindazole, a neuronal nitric oxide (NO) synthase inhibitor, reduced this transient vasodilation-implicating NO as an important participant in cerebral autoregulation. However, sustained vasodilation was not seen during the ischemic period, but this may be secondary to passive collapse of the microvessels due to slow perfusion and relatively low intravascular pressure. Despite occlusion of 4 vessels, there was residual forward flow during ischemia, which suggests that this animal model is one of incomplete global ischemia. Residual flow of plasma without erythrocytes could be seen *in vivo* in surface capillaries during the ischemia [54]. The transient arteriolar dilatation in response to tGCI was not seen in another study using a bilateral common carotid artery occlusion model in gerbils [55]. These investigators observed an initial mild arteriolar vasoconstriction in the first minute followed by a more extensive constriction beyond 1.5 minutes. These changes correlated with changes in cerebral metabolism. 

Upon reperfusion in the study by Pinard et al., blood flow could be observed in the parenchymal arterioles with significant dilatation beginning 5 minutes after unclamping of the common carotid arteries, with return to baseline arteriolar diameter after 15 minutes [54]. Another study used 10 minutes of tGCI induced in cats by a 4-vessel occlusion and systemic hypotension protocol [20]. *In vivo* imaging through a cranial window revealed persistent dilated pial microvessels upon reperfusion although CBF was reduced [20]. Overall cerebrovascular resistance was unchanged, meaning that obstruction to flow must have been present distally in the penetrating arterioles or other vessels not seen on the cortical surface [20]. However, a contrasting result was found in a tGCI model of bilateral common carotid artery occlusion in gerbils, in which the investigators did not observe vasodilation but rather found decreased diameters in both surface precapillary arterioles and capillaries during reperfusion after 15 minutes of tGCI [56]. The authors concluded that the hypoperfusion that typically occurs in tGCI is a result of increased tone in precapillary arterioles, in contrast to any conclusion that could be drawn from other studies.

Endothelial protrusions can be seen in tGCI. In a 4-vessel occlusion model of tGCI in rats with 30 minutes of ischemia, cerebral endothelial microvilli projecting into the lumen could be identified throughout the brain, and this occurred in as little as 10 minutes after initiation of ischemia [57]. The frequency of microvilli increased with increasing duration of ischemia [57]. In another study, cerebral endothelial cell microvilli were also seen after tGCI was induced by occlusion of the cardiac vessel bundle, mimicking cardiac arrest in rats [58].

### 5.2. Leukocyte-Endothelial Interactions

The preclinical studies investigating leukocyte-endothelial interactions in tGCI have had mixed results. In a 4-vessel occlusion model of tGCI in rats, the investigators studied leukocyte-endothelial interactions in pial vessels via a closed cranial window and intravital microscopy [59]. At 2 hours after an ischemic period of 20 minutes, there was no significant increase in the number of rolling or adherent leukocytes in the microvessels when compared to the control group, despite evidence of neuronal injury on histology. In another study, 30 minutes of transient forebrain ischemia was induced in gerbils by bilateral carotid artery occlusion [60]. Gerbils were treated with cyclophosphamide to decrease neutrophil count (and as a side effect, slightly decreased platelets), but this did not affect the occurrence of the no-reflow phenomenon upon reperfusion, making leukocyte plugging of small microvessels less likely as a cause of postischemic hypoperfusion. Dirnagl et al. studied tGCI in rats with bilateral common carotid artery occlusion for 10 minutes followed by 4 hours of reperfusion and found that there was a trend toward increased leukocyte rolling and adherence to the endothelium during the postischemic period [61]. Very few microvessels were plugged with leukocytes and about half of the rats demonstrated leukocyte extravasation into the parenchyma during the post-ischemic period. The transition from hyperemia to post-ischemic hypoperfusion did not reveal any obvious change in leukocyte behavior, also suggesting that leukocyte plugging would not be a major contributor to hypoperfusion in the microvasculature. In contrast, other studies have demonstrated significant leukocyte adherence to the luminal walls of the microvasculature. Ritter and colleagues found a significant increase in leukocyte rolling and adhesion in cerebral cortical venules at 30 minutes after reperfusion in a bilateral carotid artery occlusion model with induced hypotension in rats [62]. In a gerbil model of tGCI with bilateral common carotid artery occlusion for 15 minutes followed by reperfusion, there was an increase in leukocytes rolling or adhering to the venular endothelium within 3 hours of reperfusion, but no observed plugging of the capillaries, as determined by intravital fluorescence microscopy [63]. However, leukocyte-endothelial interactions had returned to baseline by 7 hours after ischemia and remained so at 12 hours and 4 days. 

The conflicting results with regard to increased leukocyte-endothelial adherence after tGCI may be related to the diversity of animal models used, the variability in the duration of ischemia and reperfusion, as well as the varied resolution of the *in vivo* microscopy equipment.

### 5.3. Blood Brain Barrier Disruption

Transient global cerebral ischemia is also believed to induce an inflammatory state that results in BBB disruption. In a bilateral carotid artery occlusion model of global ischemia in gerbils, the BBB was disrupted, as determined by extravasation of Evan's blue dye and increased brain edema [64]. Brain edema was present immediately after reperfusion although Evan's blue dye leakage was not detected until 2 hours afterwards, and both were increased 3 hours after reperfusion, which was the latest time examined. In a 4-vessel occlusion model of global cerebral ischemia in rats, BBB breakdown, as determined by leakage of labeled albumin, was greater after longer ischemia time (60 minutes of global ischemia compared to 15 or 30 minutes) [65]. The degree of associated brain edema was also dependent on the duration of the initial ischemia. In a 4-vessel occlusion tGCI model in rats, BBB breakdown occurred during the ischemic insult, as demonstrated by leakage of fluorescein dye, beginning after as little as 8 minutes of ischemia and resolving by 30 minutes after reperfusion, after a preplanned total of 15 minutes of ischemia [54]. Similar to SAH, oxidative damage to the microvessels occurs with reperfusion after tGCI. Zheng et al. demonstrated decreased activities of superoxide dismutase and glutathione peroxidase in a bilateral common carotid artery occlusion mouse model of tGCI [66]. Loss of these enzymes that protect against oxidative damage resulted in cortical microvascular endothelial damage and mitochondrial injury. The authors also found that treatment with crocin, an antioxidant, inhibited this oxidative damage and attenuated MMP-9 expression.

### 5.4. Platelet Aggregation and Microthrombosis

In a circulatory arrest model of tGCI, aggregates of platelets were identified in the intraparenchymal vessels during reperfusion after 5 minutes of tGCI [67]. Platelet aggregates increased with increasing time of reperfusion. In a 4-vessel occlusion model of tGCI in rats, thrombi could be seen *in vivo,* temporarily obstructing cortical surface arterioles and venules during the hyperemic phase after reperfusion and causing turbulent blood flow [54]. In another study, tGCI was induced by occlusion of the cardiac vessel bundle in rats for 10 minutes followed by reperfusion [58]. Microthrombi were most prominent at 3 minutes to 6 hours after reperfusion and appeared to localize in regions of relative hypoperfusion [58]. The microthrombi were not seen 7 days after tGCI in this model.

Endothelial injury occurs in tGCI which causes breakdown of the BBB, exposing portions of the basal lamina to the cerebral circulation. This promotes platelet aggregation and thrombosis. Another potential initiator of microthrombi is the relative stasis of blood during the ischemia in both SAH and tGCI—resulting in *in situ* thrombosis, although this has not been confirmed experimentally. 

## 6. Comparison of Microvascular Changes in SAH and tGCI

Although microvascular constriction is consistently demonstrated in SAH, such constriction is inconsistent during the ischemic and reperfusion phases of tGCI. This may be related to the heterogeneity in animal models utilized. However, endothelial luminal protrusions have been demonstrated in both SAH and tGCI, but the significance of this finding is unclear. Most studies that involve *in vivo* observations of microvessels typically focus on surface pial vessels, which are clearly more accessible and convenient to study. It is, however, much more difficult to assess penetrating parenchymal microvessels *in vivo*, but these vessels may be important in the pathophysiology of SAH and tGCI.

SAH and tGCI both are believed to induce inflammatory states in the brain. While less widely investigated, there does seem to be evidence that increased leukocyte adherence to the cerebral microvasculature occurs after SAH. Neutrophil adherence in tGCI has been inconsistently shown. Leukocyte rolling has also been inconsistently demonstrated in both SAH and tGCI. The no-reflow phenomenon after tGCI appears not to be directly caused by leukocyte plugging in the microvasculature.

The majority of studies investigating BBB integrity after SAH or tGCI do not use *in vivo* observation of the BBB. However, BBB disruption is consistently seen in all of these studies and appears to occur earlier after tGCI (as early as 8 minutes) compared with SAH (3 hours) [45, 54].

Platelet aggregation and presence of microthrombi in the microvessels occur after both SAH and tGCI. The models of SAH may induce some degree of tGCI, so it is difficult to determine how much of the pathophysiology after SAH is due to the subarachnoid blood itself.

## 7. Conclusions

Subarachnoid hemorrhage and tGCI share common pathophysiological changes in the microvasculature. This includes microvascular constriction during the ischemic phase, increased leukocyte-endothelial interactions, disruption of the BBB, and microvascular platelet aggregates and microthrombosis. The cerebral microvasculature may be an important target for treatments designed to reduce brain injury, although there are few such studies and limited information about the importance of the pathophysiologic processes in humans. Due to similar pathological mechanisms between these two conditions, however, it may be that treatment strategies for SAH may be applicable to tGCI and vice versa.
